# Clinical outcomes and material-selection considerations for titanium mesh and patient-specific polyetheretherketone cranioplasty: a retrospective cohort study

**DOI:** 10.3389/fneur.2026.1725628

**Published:** 2026-10-02

**Authors:** Wei Wang, Shilin Liu, Yifan Fu, Xiaoxuan Li, Minghao Xu, Xiaochan Xu, Tao Jiang

**Affiliations:** 1 Department of Emergency, The Second Affiliated Hospital of Anhui University of Traditional Chinese Medicine, Hefei, Anhui, China; 2 Department of Neurosurgery, The First Affiliated Hospital of Anhui Medical University, Hefei, China; 3 Anhui Public Health Clinical Center, Hefei, China; 4 Anhui Provincial Institute of Translational Medicine, Hefei, China

**Keywords:** cranioplasty, cranium, polyetheretherketone, postoperative complications, titanium mesh

## Abstract

**Objective:**

To compare outcomes after titanium mesh and patient-specific polyetheretherketone (PEEK) cranioplasty and to interpret the findings in the context of material-specific clinical indications.

**Methods:**

We retrospectively analyzed 99 patients who underwent cranioplasty over four years with either titanium mesh (n = 60, 60.6%) or patient-specific PEEK implants (n = 39, 39.4%). Available demographic, operative, neurological, cognitive, postoperative inflammatory, temperature, and complication data were compared. Preoperative inflammatory markers, estimated blood loss, transfusion, and standardized patient-satisfaction outcomes were not consistently available.

**Results:**

The groups differed in age and comorbidity burden (both *p* < 0.01) but not in sex, defect-diameter category, or operative duration. NIHSS, GOS, and MMSE scores did not change significantly within the titanium group. Within the PEEK group, NIHSS and GOS did not change significantly, whereas MMSE increased at 3 months (*p* = 0.005). The complication-free proportion was 41.7% in the titanium group and 64.1% in the PEEK group (unadjusted *p* = 0.029). Mean temperature during the first three postoperative days was 37.17 ± 0.36 °C in the titanium group and 36.91 ± 0.31 °C in the PEEK group (*p* < 0.01). These comparisons were unadjusted and do not establish causality.

**Conclusion:**

In this retrospective cohort, PEEK was associated with fewer recorded postoperative complications and lower mean postoperative temperature, while MMSE improved within the PEEK group. These unadjusted findings do not establish universal material superiority. Implant selection should be individualized according to defect size and geometry, anatomical location, soft-tissue condition, prior infection or implant failure, cosmetic requirements, availability, and cost.

## Introduction

In patients with a defective skull, the overall osseous protective function of the skull is compromised, leading to potential disorders of cerebrospinal fluid dynamics and cerebral hemodynamics. These disorders can manifest as neurological symptoms such as dizziness, headache, symptomatic epilepsy, depression, and memory loss (1) Cranioplasty is essential for such patients, as it not only restores the aesthetic appearance of the skull but also reduces the risk of postoperative complications and significantly alleviates intracranial hypertension symptoms (1, 18). However, there is no clinical consensus on the optimal material for cranioplasty. Commonly used materials in clinical practice include polyetheretherketone (PEEK) bone plates, titanium mesh, hydroxyapatite, porous polyethylene, biologically active materials, and autologous bone flaps (19). An ideal cranial bone defect repair material should meet the following criteria: (1) biocompatibility (strong anti-infective properties and no immunological rejection); (2) osteoconductivity (provides a framework for the growth of new blood vessels and new bone formation); (3) osteoinductivity (directly induces mesenchymal stromal cells to differentiate into osteoprogenitor cells and osteoblasts, leading to the formation of new bone tissues); (4) structural similarity to autologous bone tissue; and (5) ease of manipulation and shaping during surgery (2).

Although autologous bone flaps possess several biological advantages, postoperative resorption can necessitate secondary surgery (3). Titanium mesh is widely used because of its mechanical strength, availability, and intraoperative contourability, although imaging artifacts, thermal conductivity, palpability, exposure, and soft-tissue considerations may affect its suitability. Patient-specific PEEK implants are radiolucent and can reproduce complex cranial contours, but cost, manufacturing lead time, fixation strategy, and the possibility of infection or implant removal must also be considered (3–5).

In this retrospective cohort study, we compared postoperative outcomes after titanium mesh and patient-specific PEEK cranioplasty at a tertiary care center over four years. Rather than assuming that the materials are interchangeable, we sought to describe observed outcome differences and place them within a clinically oriented material-selection framework.

## Materials and methods

### Data collection

Between January 2019 and April 2023, patients who underwent cranioplasty at a tertiary academic center were identified. A retrospective review of medical records was conducted to collect sex, age, comorbidities, surgery duration, cranial-defect diameter, and neurological and cognitive scores preoperatively and at 3 months postoperatively (National Institutes of Health Stroke Scale [NIHSS], Glasgow Outcome Scale [GOS], and Mini-Mental State Examination [MMSE]). Available first-postoperative-day inflammatory indices (white blood cell count and percentages or counts of leukocyte subtypes), mean body temperature during the first three postoperative days, and postoperative complications (subgaleal fluid collection, subgaleal air collection, and epidural hematoma) were recorded (6). The term “subgaleal air collection” refers to postoperative air in the extracranial subgaleal space and should not be confused with pneumoperitoneum, which denotes intraperitoneal gas. Preoperative inflammatory indices, estimated blood loss, transfusion requirement, standardized patient satisfaction, defect surface area, detailed anatomical location and geometry, soft-tissue quality, previous implant history, and direct or total treatment costs were not consistently documented and therefore could not be analyzed retrospectively.

### Statistical analysis

Statistical analysis was performed using SPSS version 26.0.0 (IBM Corp., Armonk, NY). Normally distributed data were expressed as mean ± standard deviation (SD), while non-normally distributed data were presented as median and interquartile range (IQR). Group differences were assessed using t-tests for continuous variables and chi-square tests for categorical variables. A two-sided *p*-value < 0.05 was considered statistically significant. We considered multivariable logistic regression for the composite postoperative complication outcome, with age and comorbidity burden as prespecified covariates. However, comorbidity categories were incompletely classified and contained sparse cells relative to the prespecified covariates. A regression model under these conditions would be unstable and difficult to interpret; therefore, only unadjusted analyses are reported. No imputation or reassignment of outcomes was performed (see Figures 1, 2).

**Figure 1:**
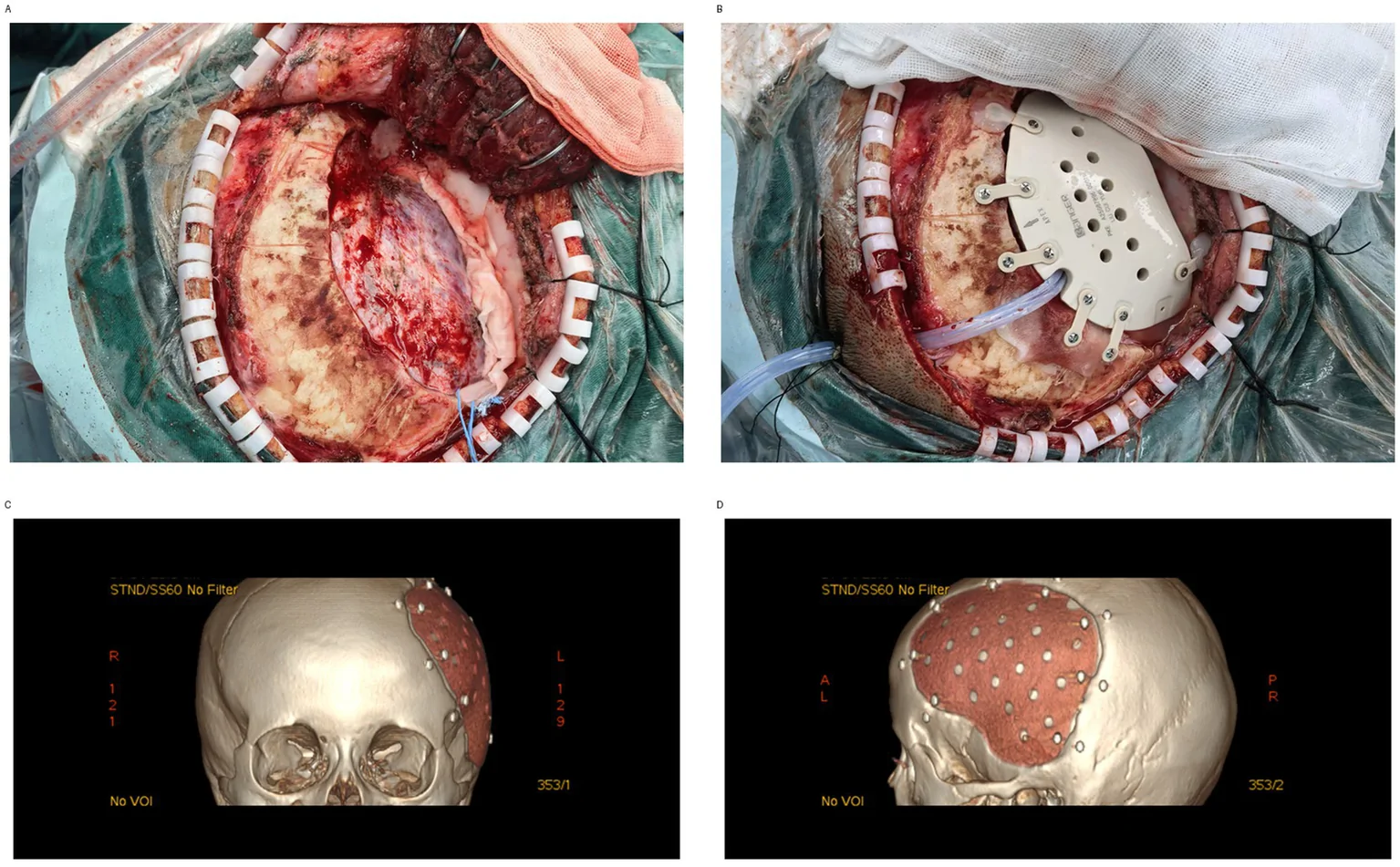
Representative patient-specific PEEK cranioplasty. **(A)** Intraoperative exposure of the cranial defect; **(B)** the PEEK implant seated and fixed to the surrounding skull; **(C)** anterior and **(D)** lateral postoperative three-dimensional CT views. The operative and CT images are representative and are not presented as a paired image series from the same patient.

**Figure 2:**
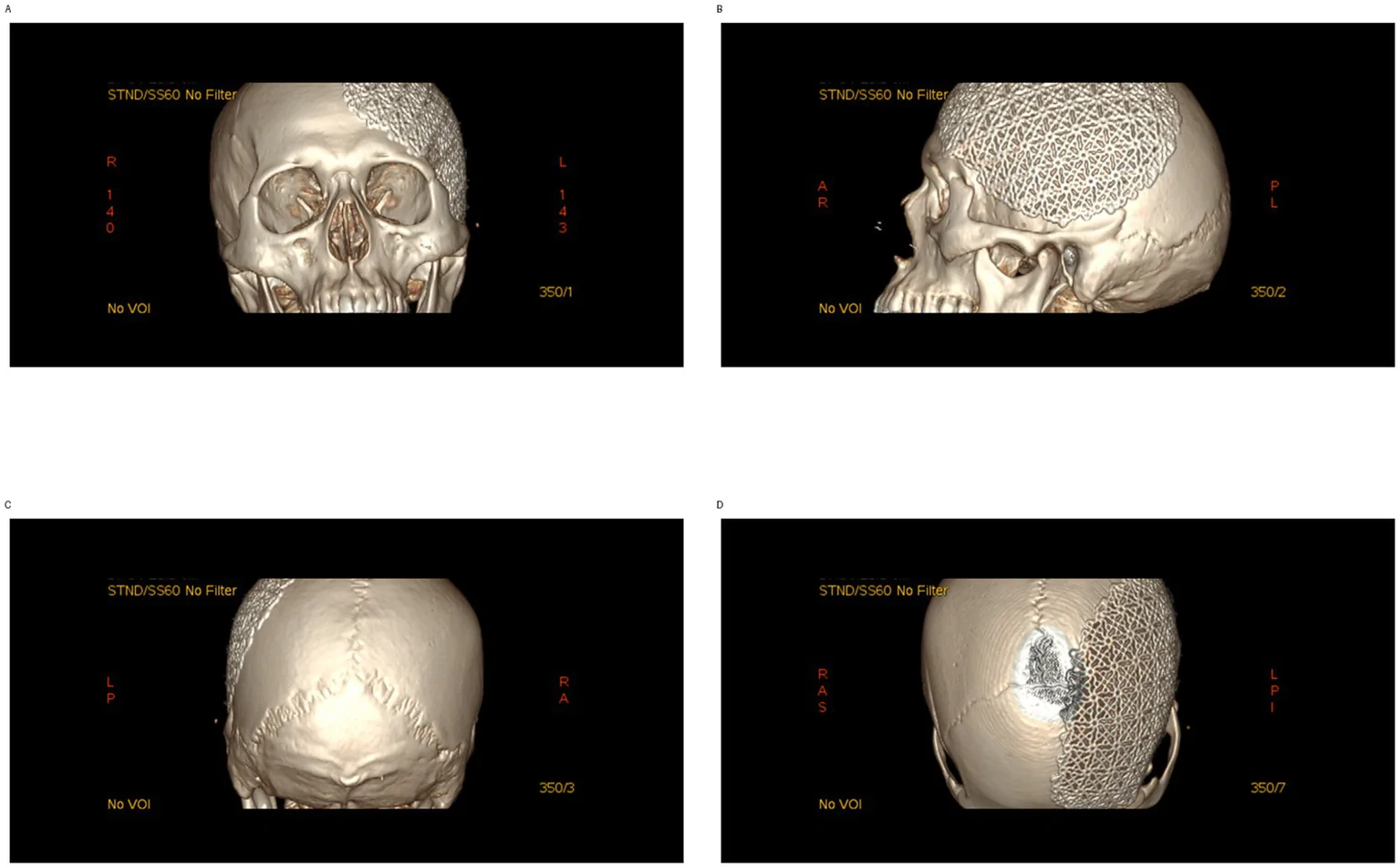
Representative de-identified postoperative three-dimensional CT views after titanium-mesh cranioplasty. **(A)** Anterior view; **(B)** lateral view; **(C)** posterior view; **(D)** superior view.

## Results

### Demographics

The groups did not differ significantly in sex distribution (*p* = 0.42), defect-diameter category (*p* = 0.27), or operative duration (*p* = 0.81) (Table 1). However, the PEEK group was younger than the titanium group (34.36 ± 15.10 vs. 47.75 ± 14.00 years, *p* < 0.01), and comorbidity distributions also differed (*p* < 0.01). These baseline imbalances were considered when interpreting the unadjusted outcome comparisons. A stable multivariable model could not be fitted because comorbidity categories were sparse and incompletely classified relative to the prespecified covariates. Consequently, an adjusted effect estimate would be unstable and is not reported.

**Table 1:** Demographics.

Variable	Total	Titanium (*n* = 60)	Polyetheretherketone (*n* = 39)	*p*-value
Total patients	99 (100%)	60 (60.6%)	39 (39.4%)	
Male	73 (73.7%)	46 (63.0%)	27 (37.0%)	0.42
Females	26 (26.3%)	14 (53.8%)	12 (46.2%)	
Age		47.75 ± 14.002	34.36 ± 15.097	<0.01
Comorbidities				<0.01
Hypertension	24	19	5	
Diabetes	3	3	0	
Hypertension combined with diabetes mellitus	1	1	0	
Hypertension combined with coronary heart disease	2	2	0	
No underlying disease	69	35	34	
Surgery duration		197.68 ± 67.68	201.62 ± 92.65	0.81
Cranial defect diameter				
<5	5 (5.1%)	4 (80.0%)	1 (20.0%)	
5 to 10	53 (53.5)	24 (45.3%)	29 (54.7%)	0.27
>10	41 (41.4%)	32 (78.0%)	9 (22.0%)	

### NIHSS, GOS, and MMSE scores

The National Institutes of Health Stroke Scale (NIHSS), Glasgow Outcome Scale (GOS), and Mini-Mental Status Examination (MMSE) were utilized to assess neurological deficits, prognosis, and cognitive function, respectively (7–9). In the titanium mesh (Ti) group, no significant differences were observed in NIHSS, GOS, and MMSE scores. In the polyetheretherketone (PEEK) group, no significant differences were observed in NIHSS and GOS scores between the preoperative and 3-month postoperative periods; however, a significant improvement was noted in MMSE scores (*p* < 0.01) (Table 2).

**Table 2:** Postoperative NIHSS, MMSE, and GOS scores.

Outcome measure	Pre-operative	Post-operative	*p*-value
Titanium			
NIHSS score	7.50 ± 5.96	7.23 ± 6.17	0.81
MMSE scores	19.97 ± 7.23	20.43 ± 744	0.73
GOS score	3.48 ± 0.60	3.58 ± 0.70	0.4
Polyetheretherketone			
NIHSS score	6.05 ± 5.07	5.21 ± 4.49	0.44
MMSE scores	24.15 ± 4.25	26.72 ± 3.47	0.005
GOS score	4.26 ± 0.91	4.41 ± 0.85	0.44

### Postoperative complications

The chi-square test was used to compare the composite occurrence of three recorded postoperative complications: subgaleal air collection, subgaleal fluid collection, and epidural hematoma. The complication-free proportion was 64.1% in the PEEK group and 41.7% in the titanium group (*p* = 0.029; Table 3). Within the titanium group, the recorded complication proportion increased across defect-diameter categories (*p* = 0.013; Table 4). The diameter-stratified PEEK counts were not retained because they were internally inconsistent with the overall PEEK complication count in Table 3 and could not be verified from the available records. Subgaleal fluid collection is a recognized outcome requiring material- and technique-specific reporting (10). Because treatment was not randomized and the groups differed in age and comorbidities, these unadjusted associations should not be interpreted as proof that implant material caused the observed differences.

**Table 3:** Postoperative complications (overall cohort comparison).

Cranioplasty material	Total	Postoperative complications	No postoperative complications	*p*-value
Titanium	60 (60.6%)	35 (58.3%)	25 (41.7%)	0.029
Polyetheretherketone	39 (39.4%)	14 (35.9%)	25 (64.1%)	

**Table 4:** Postoperative complications after grouping according to defect diameter (illustrative only).

Cranioplasty material	Complication status		Diameter <5		Diameter 5–10		Diameter > 10		*p*-value
	Postoperative complications		1 (25.0%)		11 (45.8%)		23 (71.9%)		0.013
Titanium									
	Without postoperative complications		3 (75.0%)		13 (54.2%)		9 (28.1%)		
Polyetheretherketone		Postoperative complications		0 (0.0%)		10 (34.5%)		4 (44.4%)	
		Without postoperative complications		1 (100%)		19 (65.5%)		5 (55.6%)	

Illustrative reconciliation only: the PEEK complication counts (0 + 10 + 4 = 14) and no-complication counts (1 + 19 + 5 = 25) match Table 3. These values are not recovered patient-level data and must not be used for submission without verification against the original records.

### Postoperative inflammatory indicators and body temperature

First-postoperative-day inflammatory indices did not differ significantly between groups (Table 5). Mean body temperature during the first three postoperative days was lower in the PEEK group than in the titanium group (36.91 ± 0.31 °C vs. 37.17 ± 0.36 °C, *p* < 0.01). Because corresponding preoperative inflammatory values were unavailable, changes from baseline could not be calculated; therefore, these postoperative measurements cannot establish a material-specific inflammatory response. The small absolute temperature difference should likewise be interpreted cautiously and may reflect clinical or perioperative confounding.

**Table 5:** Postoperative inflammatory indicators and body temperature.

Inflammatory indicator / Body temperature	Titanium	Polyetheretherketone	*p*-value
White blood cell count	12.19 ± 2.29	12.01 ± 2.35	0.79
Neutrophil percentage	79.98 ± 7.40	79.38 ± 7.59	0.78
Lymphocyte count	2.46 ± 2.83	1.69 ± 0.73	0.18
Eosinophil count	0.11 ± 0.28	0.02 ± 0.03	0.08
Basophil count	0.03 ± 0.06	0.02 ± 0.01	0.32
Temperature	37.17 ± 0.36	36.91 ± 0.31	<0.01

## Discussion

Titanium mesh and patient-specific PEEK implants have distinct technical profiles rather than universally interchangeable indications. Titanium mesh is strong, readily available, and can be contoured intraoperatively, but it may produce imaging artifacts and can be difficult to contour and fix across complex anatomical transitions (11–14). Patient-specific PEEK is radiolucent and can reproduce complex contours, although its cost, manufacturing time, fixation requirements, soft-tissue coverage, and susceptibility to infection or implant failure remain relevant (13–16). Accordingly, outcome comparisons should be interpreted within the clinical context that determined implant selection.

The two cohorts were similar in sex distribution, defect-diameter category, and operative duration, but differed significantly in age and comorbidity burden (Table 1). These imbalances, patient participation in implant choice, and the lack of multivariable adjustment create substantial risk of selection and confounding bias. Consequently, pooled outcome comparisons should be considered exploratory and should not be used to claim equivalent indications or causal superiority of either material.

Cranioplasty material selection is context dependent. Patient-specific PEEK can reproduce complex cranial curvature and sharp anatomical transitions and may therefore be attractive for large, irregular, frontotemporal, orbitofrontal, or cosmetically demanding defects. Titanium mesh can be contoured intraoperatively, is readily available, and may be more economical for smaller or geometrically simpler defects. Conversely, neither material is inherently infection resistant: PEEK infection and implant removal are recognized (16), while titanium may be considered in selected revision cases only after prior infection has been eradicated and soft-tissue coverage is suitable (17). These considerations were not captured in sufficient detail in our dataset and may have influenced implant allocation and outcomes.

Neurological and functional scores did not change significantly within either group, except for MMSE in the PEEK cohort (Table 2). The within-group MMSE improvement should not be attributed to PEEK itself because the study did not directly test the between-group difference in change, the PEEK cohort was younger, and recovery after cranioplasty may reflect restoration of cerebral perfusion and cerebrospinal-fluid dynamics independent of implant composition. Similarly, the lower composite complication proportion observed with PEEK is an association rather than proof of a material effect. Defect complexity, implant fit, soft-tissue condition, surgical technique, and selection of patient-specific implants may all contribute.

Postoperative inflammatory indices were similar between groups, whereas mean temperature during the first three postoperative days differed by 0.26 °C. Without preoperative inflammatory values, perioperative change cannot be assessed, and the modest temperature difference cannot be confidently assigned to the implant. Infection, tissue injury, medications, transfusion, and other perioperative factors may affect these measures. The result should therefore be regarded as hypothesis generating rather than evidence of a lower inflammatory potential of PEEK.

### Limitations and strengths of the study

This study has several limitations. First, its retrospective, single-center design, small sample, nonrandom implant selection, baseline differences in age and comorbidities, and absence of multivariable adjustment limit causal interpretation and generalizability. We considered an age- and comorbidity-adjusted logistic model for the composite complication outcome, but the comorbidity categories were sparse and incompletely classified relative to the prespecified covariates, making a multivariable model unstable and difficult to interpret; no imputed model was therefore reported. Second, the available records did not consistently include preoperative inflammatory indices, estimated blood loss, transfusion requirement, standardized patient satisfaction or cosmetic outcome measures, defect surface area, precise anatomical location and geometric complexity, soft-tissue quality, previous implant failure or infection, manufacturing details, or direct and total treatment costs. These missing variables prevented a definitive stratified indication analysis. Third, the originally supplied PEEK diameter-stratified counts were internally inconsistent with the overall PEEK complication count. The source records available for this revision did not allow us to determine whether this discrepancy arose from incomplete documentation, data-entry error, or another source-record limitation; the PEEK diameter-stratified counts and *p*-value were therefore removed rather than imputed. Fourth, complications were analyzed as a composite, and the term used for extracranial postoperative air in the original manuscript was imprecise. Fifth, follow-up was limited to 3 months for neurological and cognitive assessments and may not capture delayed infection, exposure, implant failure, or revision. Prospective multicenter studies should use prospectively standardized covariate definitions, prespecified adjustment, longer follow-up, and validated patient-reported cosmetic and satisfaction outcomes.

## Conclusion

In this retrospective cohort, PEEK cranioplasty was associated with fewer recorded postoperative complications and a lower mean postoperative temperature than titanium mesh cranioplasty, and MMSE scores improved from baseline within the PEEK group. These unadjusted findings do not demonstrate that PEEK is universally safer or superior. Titanium mesh may remain suitable for smaller or geometrically simpler defects when availability and cost are priorities, whereas patient-specific PEEK may be favored for large, irregular, frontotemporal, orbitofrontal, or cosmetically demanding reconstructions. Material selection should be individualized according to defect anatomy, soft-tissue quality, prior infection or implant failure, cosmetic goals, manufacturing requirements, and total treatment cost. Prospective, adequately adjusted studies with standardized patient-reported outcomes are needed.

## Data Availability

The original contributions presented in the study are included in the article/supplementary material, further inquiries can be directed to the corresponding author.
